# Brief Report: Social Anxiety in Autism Spectrum Disorder is Based on Deficits in Social Competence

**DOI:** 10.1007/s10803-020-04529-w

**Published:** 2020-05-14

**Authors:** J. Espelöer, M. Hellmich, K. Vogeley, C. M. Falter-Wagner

**Affiliations:** 1 Department of Psychiatry, University Hospital Cologne, Kerpener Strasse 62, 50924 Cologne, Germany; 2 Faculty of Medicine, Institute of Medical Statistics and Computational Biology, University Hospital Cologne, Kerpener Strasse 62, 50924 Cologne, Germany; 3 Department of Psychiatry, Medical Faculty, LMU Munich, Nussbaumstr. 7, 80336 Munich, Germany; 4 Institute of Medical Psychology, Medical Faculty, LMU Munich, Goethestrasse 31, 80336 Munich, Germany; 5 Department of Psychology, University of Cologne, Gronewaldstraße 2, 50931 Cologne, Germany

**Keywords:** Autism spectrum disorder (ASD), Social competence deficit, Social anxiety, Information processing deficits, Adulthood

## Abstract

This study differentially examined the relation between two clinical constructs: “social anxiety” and “social competence” in autism spectrum disorder (ASD). Employing two questionnaires (SASKO; IU), individuals with ASD (n = 23) showed increased scores of SOCIAL ANXIETY (SASKO) and of INTOLERANCE OF UNCERTAINTY (IU), compared to a non-clinical comparison group (NC; n = 25). SOCIAL ANXIETY scores were equally increased for ASD and a reference population of individuals with social anxiety disorder (SAD; n = 68). However, results showed increased SOCIAL COMPETENCE DEFICITS in ASD compared to SAD and NC groups. This study allows drawing the conclusion that social anxiety symptoms in ASD can be traced back to autism-specific deficits in social skills and are therefore putatively based on different, substantially “deeper” implemented cognitive mechanisms.

## Introduction

Deficits in social interactional skills represent core diagnostic impairments of autism spectrum disorder (ASD). UK charity “Autistica” recently identified anxiety as one of the top ten targets for autism research (Autistica 2016). Comorbidity rates of social anxiety disorder (SAD) encompassing the fear of scrutiny by other people and the fear of showing embarrassing behavior or expectation to be negatively evaluated (APA 2013) and ASD ranges across studies from 6 to 38% (Kerns et al. 2014).

Although a bi-directional relationship between ASD-related social impairments and social anxiety symptoms in ASD has been proposed (White et al. 2010, 2014; White and Roberson-Nay 2009), only very few studies have investigated social anxiety symptoms in adults with ASD (Bejerot et al. 2014; Cath et al. 2008; Kanai et al. 2011; Spain et al. 2018). Most of these show a positive relationship between autistic and social anxiety symptoms (Bejerot et al. 2014; Cath et al. 2008; Kanai et al. 2011; Spain et al. 2018). In individuals with ASD, symptoms of social anxiety might occur due to difficulties in understanding social communication and resulting social retreat. However, some symptoms of ASD might be misinterpreted as social anxiety symptoms, which represents a challenge for differential diagnostics so that particularly individuals with ASD diagnosed late in life might be prone to a misdiagnosis of SAD (Tebartz van Elst et al. 2013). The reason for this misinterpretation might be that individuals with ASD diagnosed late in life often possess high cognitive skills and have had years of developing and refining cognitive learning processes to compensate for their fundamental deficits in social communication and interaction (Lehnhardt et al. 2011, 2013). The high effort though that has to be invested for the compensation strategies can result in exhaustion, depression and social retreat, which in turn could resemble SAD (Cath et al. 2008; Davis et al. 2014). Social retreat may even be aggravated by greater self-reflecting abilities due to the awareness of personal impairments in social interactions (Bellini 2006; Kuusikko et al. 2008; Maddox and White 2015; Tyson and Cruess 2012; White et al. 2009, 2010). Finally, ASD-related core deficits in mentalizing, i.e. difficulties in inferring other’s thoughts, feelings, and intentions, may result in social distress (White et al. 2010), perceived uncertainty in social situations (White et al. 2014), and subsequently in social avoidance (Kerns and Kendall 2012; White et al. 2014) which again might be misinterpreted as SAD instead of ASD (Beidel et al. 2010; Kerns and Kendall 2012).

Notably, avoidance behaviour or social retreat and social competence deficits may be mutually dependent (Kleinhans et al. 2010; White et al. 2014) in that avoidance leads to a lack of experience and practice with social interactions and a lack of opportunities to improve social competence. In the current study, we applied the measure Social Anxiety—Social Competence Deficit Scale (SASKO; Kolbeck 2008; Kolbeck and Maß 2009). The aim of the current study was to examine the characterization of social anxiety symptoms in ASD, that can be addressed by the appropriate subscales of SASKO *anxiety of speaking and being in focus of attention* (SPEAKING) and *anxiety of being rejected by others* (REJECTION)*,* under special consideration of social competence deficits, including the SASKO subscales *interaction deficits* (INTERACTION) and *deficits in processing social information* (INFORMATION), in a group of high-functioning adults with ASD, adults with SAD, and a non-clinical (NC) comparison group.

Previous research assumed that social unpredictability and the inability to accurately interpret and grasp intentions of others in rapidly changing social interactions might overwhelm individuals with ASD (Mazefsky and Herrington 2014; Wood and Gadow 2010) and potentially relate to social anxiety as manifested in ASD. Additionally, the concept of intolerance of uncertainty (IU) has been included because it has been shown that IU represents an essential mechanism underlying the development and maintenance of anxiety in adults with ASD (Boulter et al. 2014).

Investigating this relationship is important in order to better understand social retreat in ASD and to provide guidelines for appropriate differential diagnostics and treatment in ASD.

## Method

### Participants

24 individuals with ASD and 25 NC individuals were included in the study. Additional, a reference group with social anxiety disorder (SAD; n = 68) published with the SASKO manual (Kolbeck 2008; Kolbeck and Maß 2009) was included. ASD was diagnosed according to ICD-10 (WHO 1992) criteria in the Autism Outpatient Clinic, Department of Psychiatry. The NC group was recruited from a participant database (demographic data in Table 1). Inclusion criteria were IQ ≥ 80 (WAIS-III; Wechsler 1997; WIE; Jacobs and Petermann 2007) and age 18–65 years. One participant with ASD was excluded due to incomplete filling of the questionnaires. In the final sample, the ASD group (n = 23) and the NC group (n = 25) were matched with respect to IQ and age. The SAD group showed no significant difference in age. The three groups were not matched on gender, [χ^2^(2) = 8.09, *p* = 0.018], accordingly, gender was included as a covariate in all analyses. Ethics approval was granted by the Ethics Committee of the Medical Faculty. Written informed consent was obtained before testing.

**Table 1:** Demographic information for groups

	NC (*n* = 25)		ASD (*n* = 23)		SAD (*n* = 68)	
Variables	M	SD (Range)	M	SD (Range)	M	SD (Range)
Full IQ	110.28	14.11	118.65	15.58		
Age	38.80	10.41 (23–57)	44.00	10.55 (23–58)	37.00	10.00 (22–62)

Gender	*n*		*n*		*n*	
Male	10		17		28	
Female	15		6		40	

Groups: *NC* non-clinical control, *ASD* autism spectrum disorder, *SAD* social anxiety disorder

### Instruments

The Social Anxiety—Social Competence Deficit Scale *(*SASKO) (Kolbeck 2008; Kolbeck and Maß 2009) is a German 40-item self-report measure. The total SASKO score consists of the two main scales SOCIAL ANXIETY and SOCIAL COMPETENCE DEFICITS including two subscales, respectively. The main scale SOCIAL ANXIETY is composed of the two subscales *anxiety of speaking and being in focus of attention* (SPEAKING) and *anxiety of being rejected by others* (REJECTION). The main scale SOCIAL COMPETENCE DEFICITS includes the two subscales *interaction deficits* (INTERACTION) and *deficits in processing of social information* (INFORMATION). Respondents indicated how strong each statement applies to them on a unipolar 4-point scale (“always/mostly”, “often”, “sometimes”, and “never”). The Intolerance of Uncertainty Scale (IU) (Gerlach et al. 2008) is an abbreviated 18-item German version of the original English version (Buhr and Dugas 2002; Carleton et al. 2007; Freeston et al. 1994). Ratings are made on 5-point Likert scales (“not characteristic of me at all”, “something characteristic of me”, and “very characteristic of me”).

## Results

**Fig. 1:**
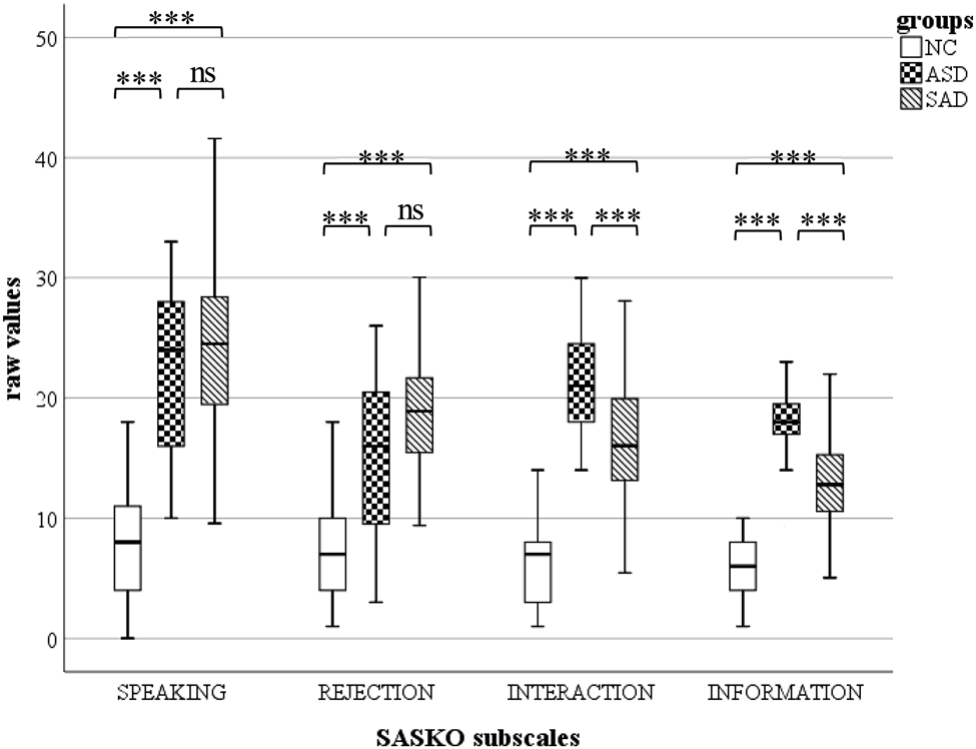
Boxplots of raw values for SASKO subscales. Groups: *NC* non-clinical control, *ASD* autism spectrum disorder, *SAD* social anxiety disorder, *SASKO* Social Anxiety—Social Competence Deficit Scale, *SPEAKING* SASKO subscale *anxiety of speaking and being in focus of attention, REJECTION* SASKO subscale *anxiety of being rejected by others*, *INTERACTION* SASKO subscale *interaction deficits*, *INFORMATION* SASKO subscale *deficits in processing of social information*. ns: *p* > 0.05. ***p ≤ .001

**Fig. 2:**
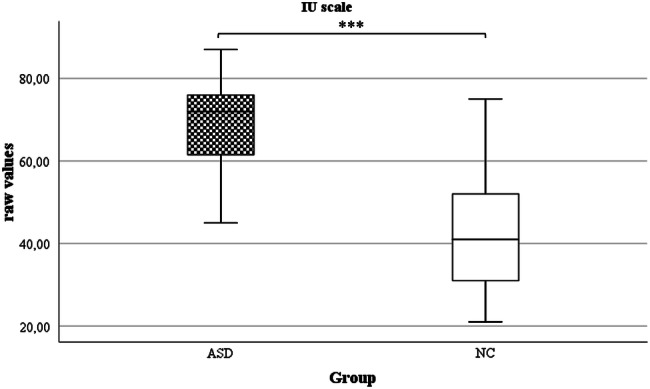
Boxplots of raw values for IU scale. Groups: *ASD* autism spectrum disorder, *NC* non-clinical control, *IU* Intolerance of Uncertainty Scale. ***p ≤ .001

Raw scores of the SASKO and the IU were analysed (descriptive data in Table 2; raw values in Figs. 1 and 2). The IU was only completed in the ASD group and the NC group. Two missing data in the SASKO were handled in accordance with the manual (Kolbeck and Maß 2009). One missing value was replaced with the mean of the scale (Maisel et al. 2016; Wigham et al. 2015). Skewness and kurtosis were within acceptable range of the absolute value of two (Gravetter 2016).

**Table 2:** Means and SDs for self-report measures by groups

	NC (*n* = 25)		ASD (*n* = 23)		SAD (*n* = 68)	
Measures	M	SD	M	SD	M	SD
SASKO total	28.52	13.35	76.57	16.93	72.18	18.33
SASKO deficit						
Subscale interaction	6.52	4.40	21.52	4.11	16.40	5.62
Subscale information	5.88	2.55	17.65	3.42	13.00	3.94
SASKO anxiety						
Subscale speaking	8.36	5.60	21.96	7.33	24.00	6.87
Subscale rejection	7.76	4.75	15.43	7.06	18.78	5.14
IU	41.56	14.63	67.09	14.72		

Groups: *NC* non-clinical control, *ASD* autism spectrum disorder, *SAD* social anxiety disorder, *SASKO* Social Anxiety—Social Competence Deficit Scale, *IU* Intolerance of Uncertainty Scale

One-way MANCOVA with one between-participant factor of Group (ASD, NC, SAD), the dependent variable Scale (SASKO TOTAL, subscales SPEAKING, REJECTION, INTERACTION, and INFORMATION), and one covariate (gender, 0–1-valued) resulted in a significant difference between the three groups on the combined dependent variables [*F*(10, 216) = 48.62, *p* < 0.001; Wilks' Λ = 0.095, partial η^2^ = 0.69]. Pairwise comparison of the ASD and the SAD group showed significantly increased scores of both subscales INTERACTION and INFORMATION in the ASD group. In both groups, values were above the clinical cut-off (cut-off raw values: INTERACTION = 10.0, INFORMATION = 9.0). No significant differences were found between the ASD and SAD group on the SASKO TOTAL scale, even though values were increased in the ASD group. In the SAD group, values of the subscales SPEAKING and REJECTION were pronounced compared to the ASD group, but no significant difference was found. However, values of both subscales were above the cut-off value (cut-off raw values: SPEAKING = 15.0, REJECTION = 13.0, SASKO TOTAL = 49.0). In the NC group, comparisons showed significantly decreased scores of all SASKO scales compared to the ASD and the SAD group (see Table 3). Results of Pearson correlations indicated that there were significant associations between all subscales, respectively, and the total score (*p* < 0.000 to *p* = 0.012). Thus, we have calculated one-way MANCOVA without SASKO TOTAL and results did not change significantly (F(8, 218) = 29.49, *p* < 0.001; Wilks' Λ = 0.231, partial η^2^ = 0.52).

**Table 3:** Pairwise comparisons for the SASKO Scales and the IU between groups

				95% CI	
Measures	Groups	*SE*	*p*	*LL*	*UL*
SASKO total^a^					
	NC: ASD	4.91	.000*	− 63.15	− 39.29
	NC: SAD	3.88	.000*	− 53.19	− 34.35
	ASD: SAD	4.13	.222	− 2.59	17.49
SASKO interaction^a^					
	NC: ASD	1.51	.000*	− 19.13	− 11.79
	NC: SAD	1.19	.000*	− 12.79	− 7.00
	ASD: SAD	1.27	.000*	2.48	8.65
SASKO information^a^					
	NC: ASD	1.03	.000*	− 14.92	− 9.91
	NC: SAD	.82	.000*	− 9.12	− 5.16
	ASD: SAD	.87	.000*	3.16	7.38
SASKO speaking^a^					
	NC: ASD	1.92	.000*	− 19.59	− 10.28
	NC: SAD	1.51	.000*	− 19.36	− 12.01
	ASD: SAD	1.61	1.000	− 4.67	3.16
SASKO rejection^a^					
	NC: ASD	1.60	.000*	− 12.31	− 4.52
	NC: SAD	1.27	.000*	− 14.13	− 7.97
	ASD: SAD	1.35	.161	− 5.91	.65
IU^a^					
	NC: ASD	4.48	.000*	− 40.69	− 18.71

*CI* confidence interval, *LL* lower limit, *UL* upper limit

Groups: *NC* non-clinical control, *ASD* autism spectrum disorder, *SAD* social anxiety disorder, *SASKO* Social Anxiety—Social Competence Deficit Scale, *IU* Intolerance of Uncertainty Scale

^a^Adjustment for multiple comparisons: Bonferroni

*p < .05

A significant group effect between the ASD and the NC group was found for the total score of the IU scale, *F*(1, 45) = 38.46, *p* < 0.001, η_p_^2^ = 0.461. No significant Levene’s test results were found for the total score of the IU scale, *F*(1, 64) = 0.05,* p* = 0.819. Pairwise comparisons indicated statistically significant differences between the ASD and the NC group (see Table 3). In the manual of the IU scale it is indicated that the scale was not standardized, but an interpretation of the individual measurements is possible by comparing them with data from the outpatient clinic (published control group: n = 651, M = 38.0, SD = 11.61; published generalized anxiety disorder group: n = 20, M = 56.7, SD = 12.49) (Gerlach et al. 2008).

## Discussion

This study aimed to characterize social anxiety in ASD and compare it to SAD and NC controls with the purpose of allowing reliable differential diagnostics and possibly suggesting tailored interventions of specific aspects of social anxiety particularly relevant in ASD. To this end, we examined deficits in social competence, defined as deficits in processing social information and interaction deficits (INFORMATION, INTERACTION) and compared it to social anxiety as accessible by the subscales of SASKO (SPEAKING, REJECTION).

SASKO TOTAL values did not differ between the ASD and the SAD group, but were clinically significant in both groups as compared to the NC group. We found SOCIAL ANXIETY values, with respect to the subscales SPEAKING and REJECTION, to be as high in the ASD group as in the SAD comparison group, confirming increased level of social anxiety in ASD (Bejerot et al. 2014; Cath et al. 2008; Kanai et al. 2011; Spain et al. 2018). Additionally, DEFICITS in SOCIAL COMPETENCE, encompassing deficits in INTERACTION and in processing social INFORMATION were significantly pronounced in the ASD group compared to individuals with SAD and the NC group.

Results suggest that social anxiety symptoms, if they occur in individuals with ASD can be traced back to the more fundamental and “deeper” layer of social competence deficits based on the idea by Karl Jaspers of a “hierarchy in the diagnostic value of symptoms” (Jaspers 1997, p. 612). According to this idea a disturbance on the “lowest plane reached by examination of the individual case decides the diagnosis.” (Jaspers 1997, p. 612). For instance, in the case of a patient with a brain injury and psychopathological symptoms resembling those of a personality disorder, the much more “fundamental” brain injury would be the dominating diagnosis but not the additional symptoms “on the surface”. In the case of ASD, one could argue that social competence deficits are fundamental whereas social anxiety symptoms are a consequence of the more fundamental disturbance of social competence skills.

Clinically, the inclusion of deficits in social skills are crucial in order to prevent misinterpretation of autistic symptoms as SAD. Social deficits in ASD might cause repeated social failure due to the perceived complexity of social interactions (Volkmar and Klin 2000), which in turn might cause supposed symptoms of social anxiety as well as social isolation (Kerns et al. 2014; Maddox and White 2015). In the case of ASD, we can make further plausible that it is the lack of social competence that leads to the avoidance of social situations rather than a disinterest in social contact (Maddox and White 2015). Indeed, many persons with ASD express a desire for social belonging to different communities (Bauminger and Kasari 2000; Bauminger et al. 2003; Maddox and White 2015; Muller et al. 2008; Tyson and Cruess 2012; White et al. 2014; Williamson et al. 2008).

Clinically elevated SOCIAL ANXIETY in both groups point out on the one hand the occurrence of social anxiety symptoms in ASD and on the other hand the problem of precise delimitation. Avoidance behavior occurs in both, individuals with ASD and individuals with SAD, but in the latter, social anxiety visible on a superficial level may cover preserved social skills (Beidel et al. 2010), whereas ASD is characterized by mentalizing deficits on a fundamental level hampering social information processing (Maddox and White 2015; White and Schry 2011). This difference is shown by significantly increased deficits in processing social information (INFORMATION) in ASD in comparison to the SAD and the NC group in the current study. In SAD, mentalizing is generally preserved, but individuals with SAD do not fully succeed to adequately evaluate social situations, which may result in dysfunctional reactions. Impaired mentalizing in ASD calls for modified interpretations of the concept of social anxiety, possibly based on Jaspers´ idea on a “hierarchy in the diagnostic value of symptoms” (Jaspers 1997, p. 612).

The results of the current study support the assumption of either atypical manifestation of SAD or co-occurring anxiety symptoms in ASD (Kerns and Kendall 2012; Kerns et al. 2014; Tyson and Cruess 2012; Wood and Gadow 2010) whereby a monodimensional model is probably not sufficient to characterize the reciprocity of social anxiety symptoms and ASD. In this context, alternatively suggested to Jaspers (1997), the psychopathological construct of so-called equifinality was proposed referring to the idea that a range of varying processes can result in the same outcome (Ollendick and Hirshfeld-Becker 2002; White et al. 2014) and thus, several anxiety symptoms appear similar, but differ in a subtle way (Kerns et al. 2014). SAD might arise from a multifaceted spectrum of etiological factors during development (Ollendick and Hirshfeld-Becker 2002; White et al. 2014) and has been linked rather to the cognitive capacity for social evaluative efforts and, moreover, to temperamental factors that represent a deeply entrenched personal characteristic (Neal and Edelmann 2003; Tyson and Cruess 2012). ASD as a pervasive developmental disorder might be better explained by a “deeper layer” of mentalizing disturbances occurring on a more profound level of social information processing. Particularly in adulthood, SAD as an acquired disorder, seems to be more likely to be described as a more superficial layer.

Research suggests less pronounced cognitive components of anxiety in ASD than in individuals with SAD (Maddox and White 2015). By contrast, in SAD, fear of negative evaluation referred to one’s own self and the cognitive component was highlighted, as well as temperamental factors (Maddox and White 2015; Neal and Edelmann 2003; Tyson and Cruess 2012; White and Schry 2011). Individuals with ASD may worry about how their own behavior affects others instead of expecting negative evaluations of their own self which again might describe the surface level. White et al. (2014) assumed an association between perceived uncertainty in social situations and mentalizing deficits but without any concern of negative evaluations of one’s own self. In ASD, fear of negative evaluation might rather affect the worry about uncertainty in social situations. Present results support this assumption by representing increased IU as well as pronounced social competence deficits in ASD. Likewise, the inability to endure uncertainty might cause social avoidance, which limits opportunities to acquire and practice social skills and to improve interpersonal communication abilities (Rubin and Burgess 2001).

This assumption of a different manifestation of social anxiety in ASD based on a more fundamental deficit could support especially the process of differential diagnosis and may possibly also enrich the development of specific psychotherapeutic interventions. Spain et al. (2017) suggest that cognitive and behavioral interventions have shown success in individuals with ASD and SAD. However, consistent with results of the current study, modified or combined interventions focusing on deficits in processing social information, emotional literacy, and impairments in social skills were recommended. Furthermore, individuals with ASD and social anxiety symptoms may benefit from continuous period of treatment as well as the opportunity to practice social skills in real-life situations (Spain et al. 2017). In addition, the SASKO instrument may serve as an important tool in the improvement of differential diagnostics.

In conclusion, individuals with ASD show a level of SOCIAL ANXIETY comparable to individuals with SAD. Nevertheless, decreased SOCIAL COMPETENCE and pronounced deficits in processing social information (INFORMATION) represent specific factors associated with social anxiety in ASD and suggest a more fundamental disturbance compared to SAD as a possible indicator of differential values of symptoms for diagnostics (Jaspers 1997). Social retreat might additionally aggravate social competence deficits throughout development.

## Supplementary Note

In addition, a further group of outpatients with ICD-10 axis-I diagnoses but without ASD was tested (n = 20). Due to the diagnostic heterogeneity of this group, statistical comparison is not readily generalizable.
